# Intra-urban variability of the intake fraction from multiple emission sources

**DOI:** 10.1016/j.apr.2018.05.003

**Published:** 2018-11

**Authors:** Piotr Holnicki, Andrzej Kałuszko, Zbigniew Nahorski, Marko Tainio

**Affiliations:** a Systems Research Institute, Polish Academy of Sciences, Warsaw, Poland; b Warsaw School of Information Technology (WIT), Warsaw, Poland; c UKCRC Centre for Diet and Activity Research (CEDAR), MRC Epidemiology Unit, University of Cambridge, UK

**Keywords:** Emission field, Pollution dispersion model, Concentration assessment, Population exposure, Intake fraction assessment

## Abstract

**Background:**

Ambient air pollution and associated adverse health effects are among most acute environmental problems in many cities worldwide. The intake fraction (*iF*) approach can be applied for evaluating the health benefits of reducing emissions, especially when rapid decisions are needed. Intake fraction is a metric that represents emission-to-intake relationship and characterizes abatement of exposure potential attributed to specific emission sources.

**Aim:**

In this study, the spatial variability of *iF* in Warsaw agglomeration, Poland, is discussed.

**Methods:**

The *iF* analysis is based on the earlier air quality modeling results, that include the main pollutants characterizing an urban atmospheric environment (SO_2_, NOx, PM_10_, PM_2.5_, CO, C_6_H_6_, B(a)P, heavy metals). The annual mean concentrations were computed by the CALPUFF modeling system (spatial resolution 0.5 × 0.5 km^2^) on the basis of the emission and meteorological data from year 2012. The emission field comprised 24 high (power generation) and 3880 low (industry) point sources, 7285 mobile (transport) sources, and 6962 area (housing) sources.

**Results:**

The aggregated *iF*s values are computed for each emission class and the related polluting compounds. Intra-urban variability maps of *iF*s are attributed to an emission sources by emission category and pollutant.

**Conclusions:**

Intake fraction is shown as a decision support tool for implementing the cost-effective emission policy and reducing the health risk of air pollution.

## Graphical abstract

**Figure:**
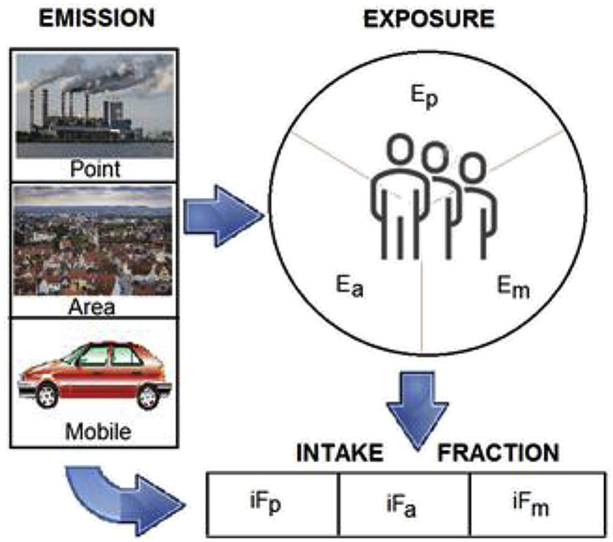

## 1 Introduction

Intake fraction (*iF*) characterizes the emission-to-exposure relationship (Bennett et al., 2002a). It is defined as the ratio of the mass of pollutant inhaled by the exposed population and the mass of pollutant emitted by a given source or by a specified category of emission sources (Levy et al., 2002; Stevens et al., 2007; Tainio et al., 2010; Lamancusa et al., 2017). It depends on several factors, such as (Marshall and Nazaroff, 2006) the exposed population size and its spatial density, the distance between the source and the receptor domains, the meteorological conditions controlling pollution dispersion, the pollutant persistence, the urban morphology (Zhou and Levy, 2008), and the chemical and physical transformation (secondary pollutant formation). On the other hand, *iF* is attributed to the unit emission because its value is normalized. Intake fraction is dimensionless, often expressed in [ppm], where 1 ppm means 1 mg inhaled for 1 kg emitted (Bennett et al., 2002b; Lamancusa et al., 2017; Apte et al., 2012).

Intake fraction is an important tool in life cycle analysis and risk assessment (Bennett et al., 2002b; Humbert et al., 2011) or in decision making process, when emission abatement strategy is considered (Marshall et al., 2005; Marshall and Nazaroff, 2006; Stevens et al., 2007), or where the population weighted intake is directly transferred to the related health effects. Given *iF*s and the emission rates for each source, solutions can be optimized, e.g. by the scenario or cost effectiveness analysis. Intake fraction plays a similar role as the transfer matrix of the emission-to-concentration relation which is a key factor in the decision problem involving reduction of pollutant concentration.

In the previous studies *iF*s were assessed for different types of emission sources and pollutants like power plant point sources (Levy et al., 2002; Zhou et al., 2003; Lamancusa et al., 2017), domestic combustion area sources (Taimisto et al., 2011; Apte et al., 2012; Tainio et al., 2014) or road transport sources (Marshall et al., 2003, 2005; Greco et al., 2007a,b; Apte et al., 2012; Du et al., 2016). Most of these studies estimated *iF*s aggregated over multiple individual sources, such as *iF* for all traffic related emission sources in a given study area (Apte et al., 2012). Only few studies considered intra-urban variation of *iF* (e.g. Tainio et al., 2014; Greco et al., 2007b), meaning variation of *iF*s among source areas in the considered domain. Emission mitigation policies can be spatially targeted, particularly for linear and area sources, such as traffic and domestic combustion. Knowing the intra-urban variation of *iF* and the emission volumes, the sources can be identified where emission reduction gives best population health effects.

In this study, the intra-urban *iFs* for Warsaw agglomeration are estimated, attributed to main emission source categories. Emission sources located outside Warsaw substantially contribute to concentrations of some pollutants (e.g. particulate matter), observed in the city (EEA, 2012; Tainio et al., 2014; Holnicki et al., 2017a). In this study, we assess how the external sources affect the *iF*s for the main pollutants.

Analysis of *iF* spatial distribution is based on the air quality modeling results for Warsaw, using the emission and meteorological data for the year 2012 (WIOŚ, 2012; Holnicki et al., 2017a). The computed concentration fields are integrated with population density, to calculate *iF*s and exposures for the pollutants considered, for different emission categories. The impact of the local and external emission sources is considered.

## 2 Materials and methods

### 2.1 The study area

**Fig. 1:**
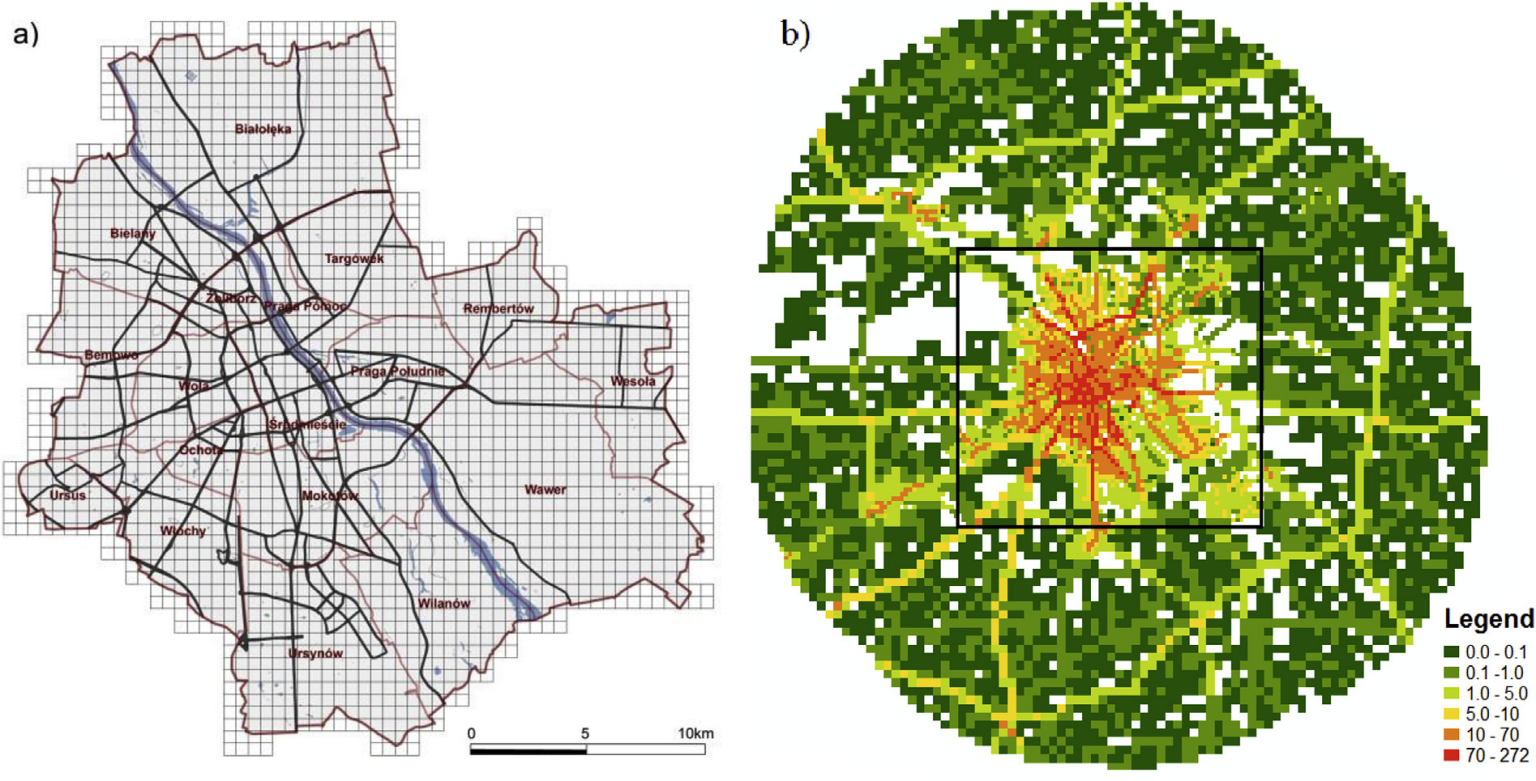
The study domain: (a) discretization of the receptor area, (b) the total emission area (PM_10_ emission in [g/s*10^8^]).

The study area, about 520 km^2^ within the administrative borders of Warsaw, Poland, and the total population of 1 715 517 inhabitants (GUS, 2014; Holnicki et al., 2017a), is shown in Fig. 1a. Air quality in Warsaw is mainly determined by two categories of emission sources, the domestic combustion and the traffic. These are the principal sources of particulate matter (PM), including its fine fraction – PM_2.5_, which is mainly responsible for air pollution-associated mortality (Wang et al., 2016; WHO, 2016; Holnicki et al., 2017b). The coal combustion is the main energy source (electricity/heat) in Poland (85%). Coal is also used for domestic heating in the peripheral districts of Warsaw as well as in the neighboring municipal areas, while the district heating system covers the central part of the city. Road transport, due to high and steadily increasing traffic intensity in Warsaw (WIOŚ, 2012), is another emission category influencing air quality. It contributes significantly to the total PM pollution, being at the same time the main source of NOx, CO and C_6_H_6_ pollution.

### 2.2 Pollutants and emissions data

The pollutants considered in this study are sulfur- and nitrogen oxides (SO_2_, NO_X_), carbon monoxide (CO), benzene (C_6_H_6_), particulate matters PM_10_ and PM_2.5_ (including primary and re-suspended fractions, and sulfate and nitrate aerosols, ${\text{SO}}_{4}^{=}$, ${\text{NO}}_{3}^{-}$), and some heavy metals (Pb, As, Cd, Ni), as well as benzo(a)pyrene B(a)P. The composition of pollutants, their spatial distribution, and their maximum values also reflect the specific structure of the local emission field (the highly emitting mobile sources in central districts and the area sources in the peripheries). All emission sources are divided into four categories: high point sources (energy sector), other point sources (industry), area sources (residential heating), line sources (road transport).

To assess the impact of the external sources, we enlarge the emission domain to be much wider than the administrative domain of Warsaw, which however remains the sole receptor domain. Hence, the total emission field covers the Warsaw area and the surrounding belt of approximately 30 km width, as shown in Fig. 1b (total emission domain). Within this domain, the rectangle area of the close vicinity of Warsaw is indicated (Fig. 1b), which includes the city in the administrative border (Warsaw only). The total emission domain covers 24 high point sources (energy generation), 3880 low point sources (industrial plants), 6963 area sources (residential combustion), 7286 line sources (road traffic, aggregated in the grid cells).

The point sources are localized by coordinates. The area and line sources are defined as grid emission squares, 0.5 km × 0.5 km inside Warsaw (Fig. 1a), and coarser resolution (1 km × 1 km) in the surrounding belt (Fig. 1b). The local city areas within this belt are represented by the nested fine resolution grid.

### 2.3 Dispersion modeling

A multi-pollutant analysis of the intra-urban variability of the intake fraction is based on modeling results for the year 2012 and the population density data for Warsaw (EEA, 2009; GUS, 2014; Holnicki et al., 2017a). This study updates the earlier modeling work that used emission and meteorological data from year 2005 in the grid 1 km × 1 km (Tainio et al., 2014). The annual mean concentrations were computed by CALMET/CALPUFF modeling system (Scire et al., 2000), with the spatial domain resolution 500 m × 500 m. Meteorological fields were obtained using the WRF model (NCAR, 2008), and assimilated to the final discretization grid by the CALMET preprocessor. The air pollution results were compiled as a sequence of 1-h episodes (8785 time steps) which cover the year under question. Finally, the annual average concentrations of the main pollutants were calculated at each fictitious receptor point located in the centers of all grid cells. For more details on air quality modeling and validation of the modelled results with measurements see (Holnicki et al., 2016).

### 2.4 Population weighted exposures and intake fractions

**Fig. 2:**
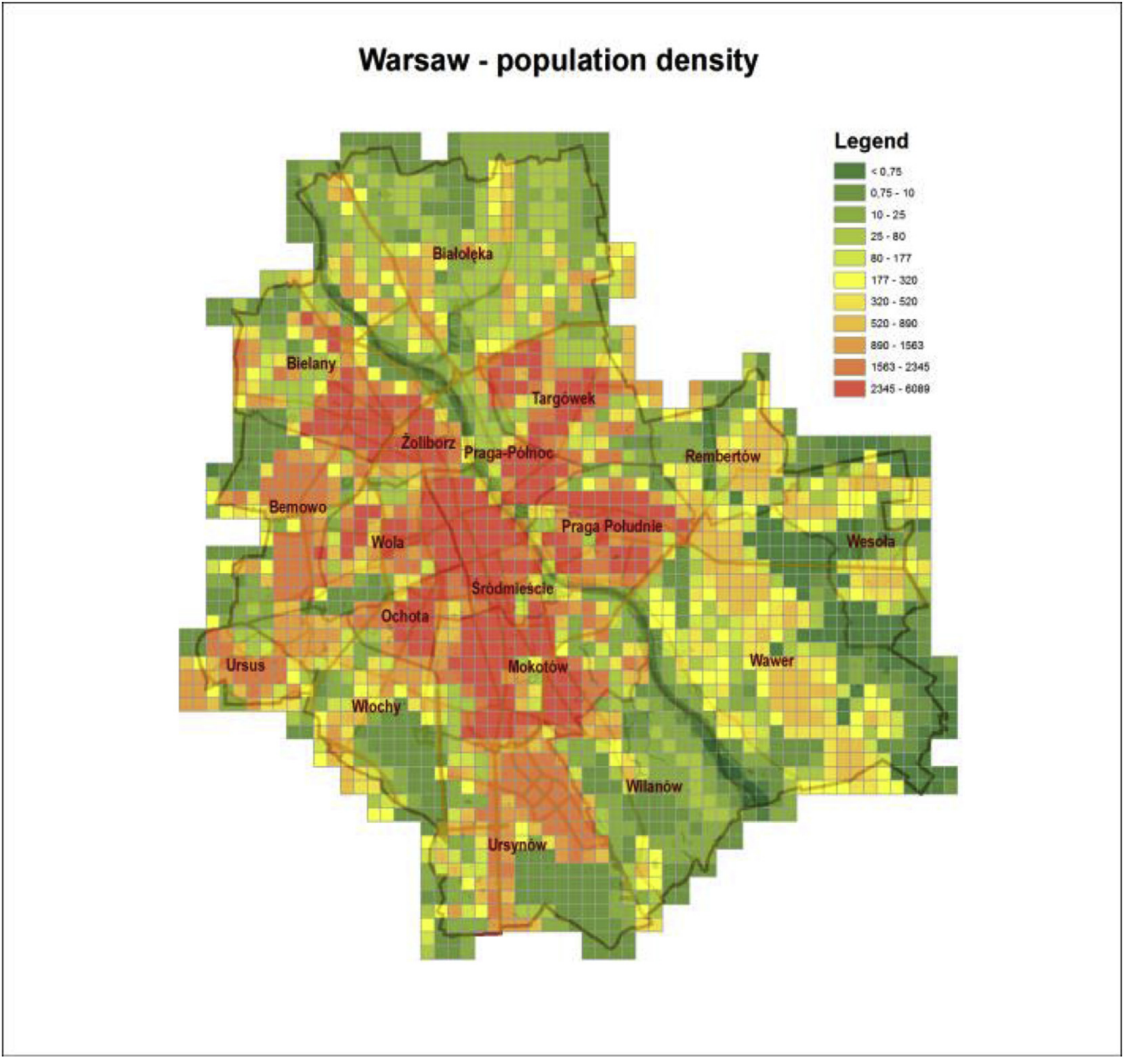
Population density map for Warsaw [persons per 0.25 km^2^] (Holnicki et al., 2017b).

Fig. 2 presents the population density map for Warsaw (Holnicki et al., 2017b), which was compiled on the basis of the home addresses for the same grid as that used in the air quality model. Movement of people was not taken into account.

Besides the intake fraction, population weighted exposure to a selected pollutant*,* $E_{i,k}$ [μg/m^3^], can be attributed to a specified emission source. It quantifies an adverse environmental impact of the source, which can be also useful when emission reduction policy is considered. Its value can be calculated as follows:

(1) $${\text{E}}_{\text{i},\text{k}}=\frac{1}{\text{Pop}}\sum_{\text{j}}{\text{C}}_{\text{i},\text{j},\text{k}}\cdot {\text{Pop}}_{\text{j}}$$

where ${\text{C}}_{\text{i},\text{j},\text{k}}\;$ is the concentration of the pollutant [μg/m^3^] originating from *i*-th source and measured at *j*-th receptor element, ${\text{Pop}}_{\text{j}}$ is the population of *j*-th receptor element [number of people in 0.25 km^2^ cell], Pop is the total population (1 715 517 assumed), *i* is the emission source's index within the emission category, *j* is the receptor's index, and *k* is the index of the pollutant.

By definition, intake fraction is a standardized measure, calculated per unit emission, which quantifies the sensitivity of environmental impact to variation of the source's emission. The *iF* value of a single source (attributed to specific *i*-th emission source and *k*-th pollutant) can be computed according to the formula:

(2) $${\left(\text{iF}\right)}_{\text{i},\text{k}}=\frac{\text{BR}}{{\text{Q}}_{\text{i},\text{k}}}\sum_{\text{j}}{\text{C}}_{\text{i},\text{k},\text{j}}•{\text{Pop}}_{\text{j}}=\;\;\frac{\text{BR*Pop}}{{\text{Q}}_{\text{i},\text{k}}}{\text{∗}\;\text{E}}_{\text{i},\text{k}}$$

where ${\text{Q}}_{\text{k},\text{j}}$ [g/s] is the emissions rate, BR is the breathing rate (20 m^3^/day/person or ∼0.00021 m^3^/s/person) as applied in (Bennett et al., 2002b; Loh et al., 2009; Du et al., 2016), and *i*, *j*, *k* as before. The calculated (*iF*)_i,k_ refers to the amount of a pollutant inhaled within the specified domain, that in our case is a grid cell.

To quantify the relation between the total emission volume and the intake of a pollutant, aggregated values of the *E* and *iF* metrics for a specific emission category (high point, other point, area, and line sources) are used. The population average (aggregated) exposure that quantifies the total impact from all sources of the considered emission category for *k*-th pollutant, is calculated according to the formula:

(3) $${\text{E}}_{\text{k}}=\frac{1}{\text{Pop}}\sum_{\text{j}}{\text{Pop}}_{\text{j}}\;\left(\sum_{\text{i}}{\text{C}}_{\text{i},\text{j},\text{k}}\right)$$

where $E_{k}$ is the total exposure to the selected pollutant [μg/m^3^], and the rest of the symbols is defined as in equation (1).

The aggregated intake fraction, ${\left(iF\right)}_{\text{k}}$, for *k*-th pollutant is now calculated as follows:

(4) $${\left(\text{iF}\right)}_{\text{k}}=\frac{\text{BR}}{{\text{Q}}_{\text{k}}}\sum_{\text{j}}{\text{Pop}}_{\text{j}}\left(\sum_{\text{i}}{\text{C}}_{\text{i},\text{j},\text{k}}\right)=\frac{\text{Pop}\cdot \text{BR}}{{\text{Q}}_{\text{k}}}{\text{E}}_{\text{k}}\;\text{and}\;{\text{Q}}_{\text{k}}=\sum_{\text{i}}{\text{Q}}_{\text{i},\text{k}}$$

where ${\text{Q}}_{\text{k}}$ is the total pollutant emission within the category, and the rest is as in equation (2).

The exposure and the intake fractions for the secondary pollutants, ${\text{SO}}_{4}^{=}$ and ${\text{NO}}_{3}^{-}$, are also computed according to equations (3), (4), using the estimated ${\text{SO}}_{4}^{=}$ and ${\text{NO}}_{3}^{-}$ concentrations. The intake fractions of the above pollutants are defined as the mass of the secondary pollutant inhaled per unit of the precursor emission, calculated on the basis of the atomic masses for primary (denominator) and the secondary (numerator) constituents, as in (Tainio et al., 2014; Levy, 2016; Lamancusa et al., 2017).

## 3 Results and discussion

### 3.1 Aggregated iFs for emission categories

Table 1 presents the aggregated intake fractions ${\left(iF\right)}_{\text{k}}$ and the related total emissions ${\text{Q}}_{\text{k}}$, computed for each emission category and all pollutants discussed in this study for Warsaw only receptor area. Generally, the aggregated *iF*s characterize the assigned emission category: being low for the high point sources and increase successively for the low point, area, and line sources, respectively. The low *iF*s for the high point sources are mainly due to high stacks of the power plants (50–300 m) in this category, due to which most of the emitted pollutants are transported outside the study area. The *iF*s for the low point sources are higher, as the stacks are significantly lower and larger fraction of pollutants stay within the study area. The changes of the *iF* estimates (between the pollution types) for the main pollutants in both categories are very small: within 1–2 ppm for high and 2–4 ppm for low point sources. This refers to the primary pollutants, as for the secondary particles the values are about two orders of magnitude lower due to long time of their formation, so that the limited modeling domain is not sufficient to capture them. The differences related to *iF*s for heavy metals are due to low number of sources emitting these pollutants.

**Table 1:** Emission, Q [g/s] ([mg/s] for As, Cd, Ni, BaP) and the aggregated intake fraction, *iF* [ppm] for the main emission categories and pollutants, considered in the total domain (PPM10, PPM 2.5 – primary pollution; PPM 10_r, PPM 25_r – re-suspended by traffic; PM10, PM 25 – total, including secondary components).

	High point		Low point		Area		Line		Total	
	Q	iF	Q	iF	Q	iF	Q	iF	Q	iF
SO2	358	**0,74**	50,9	**2,03**	221	**5,95**	52,4	**13,20**	682	**3,40**
SO4	0,00	**0,01**	0,28	**0,03**	2,97	**0,26**	0,96	**0,81**	4,21	**0,15**
NOx	256	**0,60**	44, 8	**3,80**	138	**6,02**	715	**13,92**	1154	**8,48**
NO3	0,00	**0,03**	0,45	**0,18**	3,27	**0,08**	0,00	**0,60**	13,8	**0,34**
PPM10	22,7	**0,89**	21,5	**3,99**	531	**6,81**	58,3	**16,70**	633	**7,17**
PPM10_r							238	**20,15**	238	**20,15**
PPM25	7,35	**1,01**	9,65	**4,08**	416	**6,72**	39,4	**16,55**	478	**7,14**
PPM25_r							31,5	**21,91**	31,5	**21,91**
PM10	22,7	**1,19**	22,2	**4,07**	535	**6,85**	297	**20,10**	878	**10,95**
PM25	7,35	**1,93**	10,4	**4,26**	421	**6,77**	72,0	**21,53**	511	**8,49**
CO	61,0	**0,78**	69,4	**2,62**	403	**6,43**	3347	**22,77**	3880	**20,34**
C6H6	178	**0,57**	17,4	**2,68**	0,02	**7,36**	16,2	**22,63**	212	**2,43**
Pb	0,00	**1,30**	0,01	**5,39**	0,33	**7,29**	0,18	**21,08**	0,52	**11,77**
As	1,04	**1,18**	0,48	**2,80**	35,8	**7,28**		** **	37,3	**6,75**
Cd	0,35	**0,77**	1,87	**9,08**	52,3	**7,28**	1,02	**3,52**	55,5	**6,94**
Ni	26,6	**0,86**	7,35	**5,62**	164	**7,28**	22,3	**15,65**	221	**7,06**
BaP	3,27	**1,41**	2,45	**2,31**	61,2	**6,22**	5,75	**12,57**	72,7	**6,15**

The average *iF* values are greater for the area emitters, with the values of 6–7 ppm for the main primary pollutants. Similarly, for the line sources the average *iF* was about 20 ppm and the range 14–21 ppm. The differences between the aggregated *iF*s for a pollutant (as observed, e.g. for heavy metals) result from different sets of active emission sources and their locations regarding specific reception cell.

A source of the road transport emission consists of the street segments in each emission source, where high emissions usually coincide with densely populated districts of the city. Moreover, every emission cell is at the same time the most affected receptor, so the dispersion of pollutants has relatively lower impact for *iF*s. This results in high *iF* values for traffic emissions.

It follows from Table 1 that the area and line sources have the greatest impact on *iF* estimates in Warsaw. The total number of emitters in each of them is about 7000, but most of the distant sources impact negligibly the receptor domain exposure. Results presented in Table 2 illustrate how the aggregated *iF* estimates for the above mentioned two categories depend on change of the emission domain. The table depicts a comparison of the *iF*s for three cases, where the emission field includes the total domain (Fig. 1b), the Warsaw + close vicinity domain (rectangle area in Fig. 1b), and the Warsaw only domain, inside the administrative border. The distant sources, which comprise about 50% of the total emitters in each class, only slightly contribute to the *iF* estimates (with the exception of the typical, transportation related PM, CO, C_6_H_6_), that results in low aggregated *iF* estimates. Much more significant is the impact of the sources located in Warsaw + close vicinity, while the emitters in Warsaw only domain definitely prevail. Here again the impact of the mobile emission is the greatest, with a significant contribution of the area emission (individual residential heating). A rather abnormal *iF* pattern observed for Cd follows from the fact, that only 11 minor emission sources are located in Warsaw, and the major emission sources are in its close vicinity.

**Table 2:** Comparison of the aggregated *iF*s [ppm], depending on the emission domain for the area and line source categories.

Emission	Nmb. of sources	SO_2_	NOx	PM_10_	PM_25_	CO	C_6_H_6_	BaP	Ni	Cd	Pb
**Area sources**											
Total domain	6963	5,95	6,02	6,85	6,77	6,43	7,36	6,22	7,28	7,28	7,29
Warsaw + close vicinity	2321	11,60	11,43	9,35	9,23	12,16	13,66	11,85	9,96	13,67	13,67
Warsaw only	1165	28,66	28,02	30,46	30,36	30,58	31,15	30,25	30,95	30,96	30,96
**Line sources**											
Total domain	7286	13,19	12,12	20,10	21,53	22,77	22,63	12,57	15,65	3,52	21,08
Warsaw + close vicinity	2765	20,74	19,19	29,76	29,16	29,68	29,66	17,98	22,63	6,87	27,88
Warsaw only	1828	25,96	24,40	36,94	38,80	35,70	34,67	21,34	26,93	3,28	33,02

### 3.2 Intra-urban iF variability for single sources

**Fig. 3:**
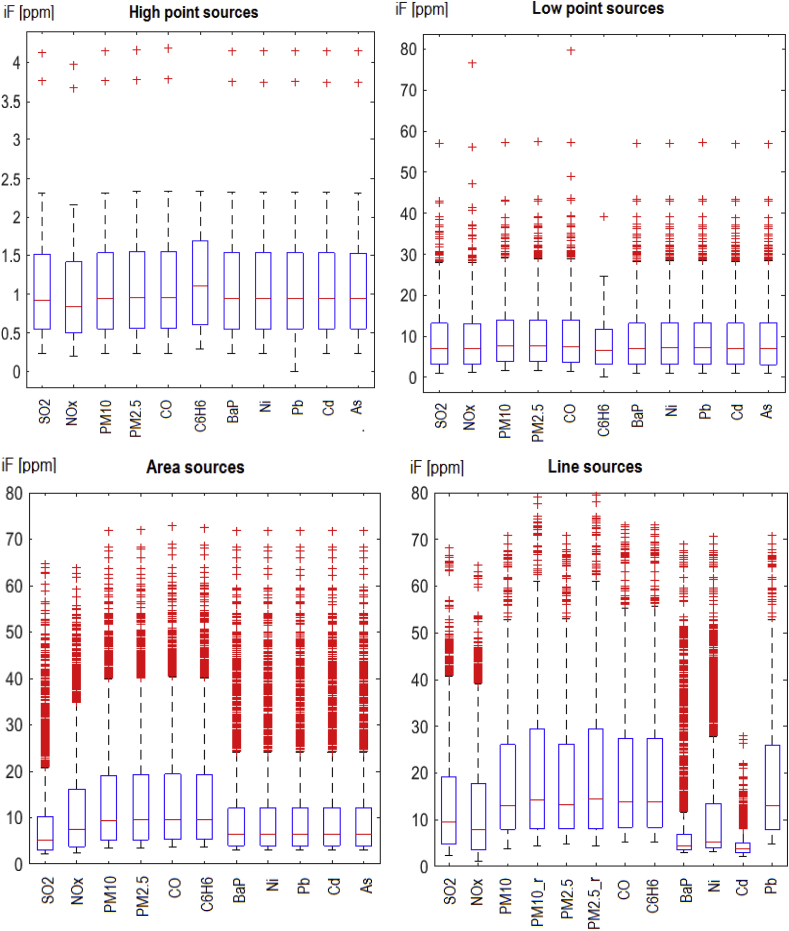
Distribution of the intake fractions by the source categories and pollutants: the high and low point sources (top) and the area and line sources (bottom). The horizontal lines on the box indicate the 25th, 50^th^ (median), 75th percentiles, while the whiskers show the 5th and 95th percentiles. The height of the box indicates the interquartile range (abbreviation IQR used in the text), limited by the 25th and 75th percentiles of distribution.

Fig. 3 presents box plots for intra-urban distributions of the *iF* estimates attributed to single sources in the four emission categories for different pollutants. The box plots for the point sources (Fig. 3a) comprise all emitters within this category. The variability of the high point emitters *iF*s, which is within the range of 0.2–4.2 ppm (min – max) and with the interquartile range (IQR) of 0.6–1.8 ppm, is relatively low, as these sources (24 in total) are generally homogeneous as to the emission parameters and all located in the Warsaw + close vicinity domain. For the low point sources, which are about 3000 in total, the variability range is 0.1–57 ppm, with IQR 3–14 ppm, for the basic pollutants. The only exceptions are NOx and CO, where the maximum *iF* values of 76.6 ppm and 79 ppm, respectively, are connected with a very specific emission source, which emits only two mentioned above components. The emission rates of this source are very low, but it has a low stack height (7 m) and is located in a densely populated central district. This provides much higher *iF* than for the rest of the sources.

In the case of the area and the mobile sources (Fig. 3b) the presented plots incorporate 2500 (for each case) emission sources with the highest *iF*s, from about 7000 active emission sources. The *iF* variability for the area sources is within the range 0–72 ppm and IQR within 4–20 ppm for PM/CO/C_6_H_6_, and for the line sources within the range 0–80 ppm and IQR within 6–30 ppm for PM, with the maximum of 80 ppm for the re-suspended (due to the road traffic) particulate matter, PM_10_r_ and PM_2.5_r_.

**Fig. 4:**
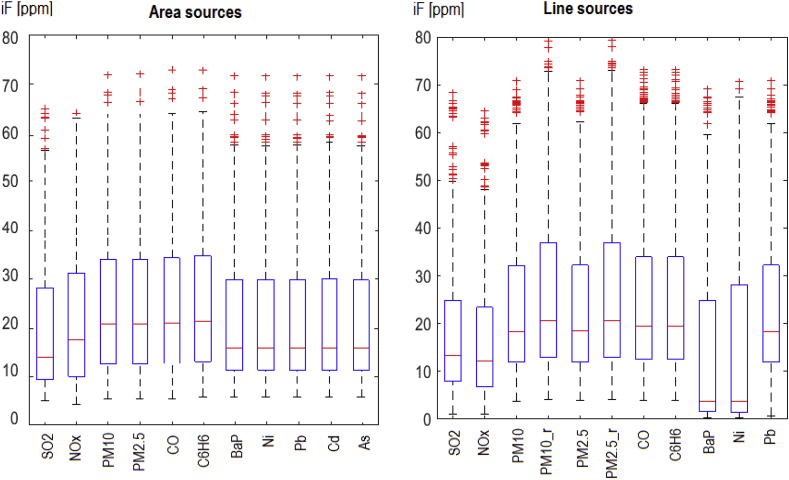
Distributions of the elementary *iF*s for the area (left) and the line (right) sources, when the intra-urban emission sources (only) are considered.

**Fig. 5:**
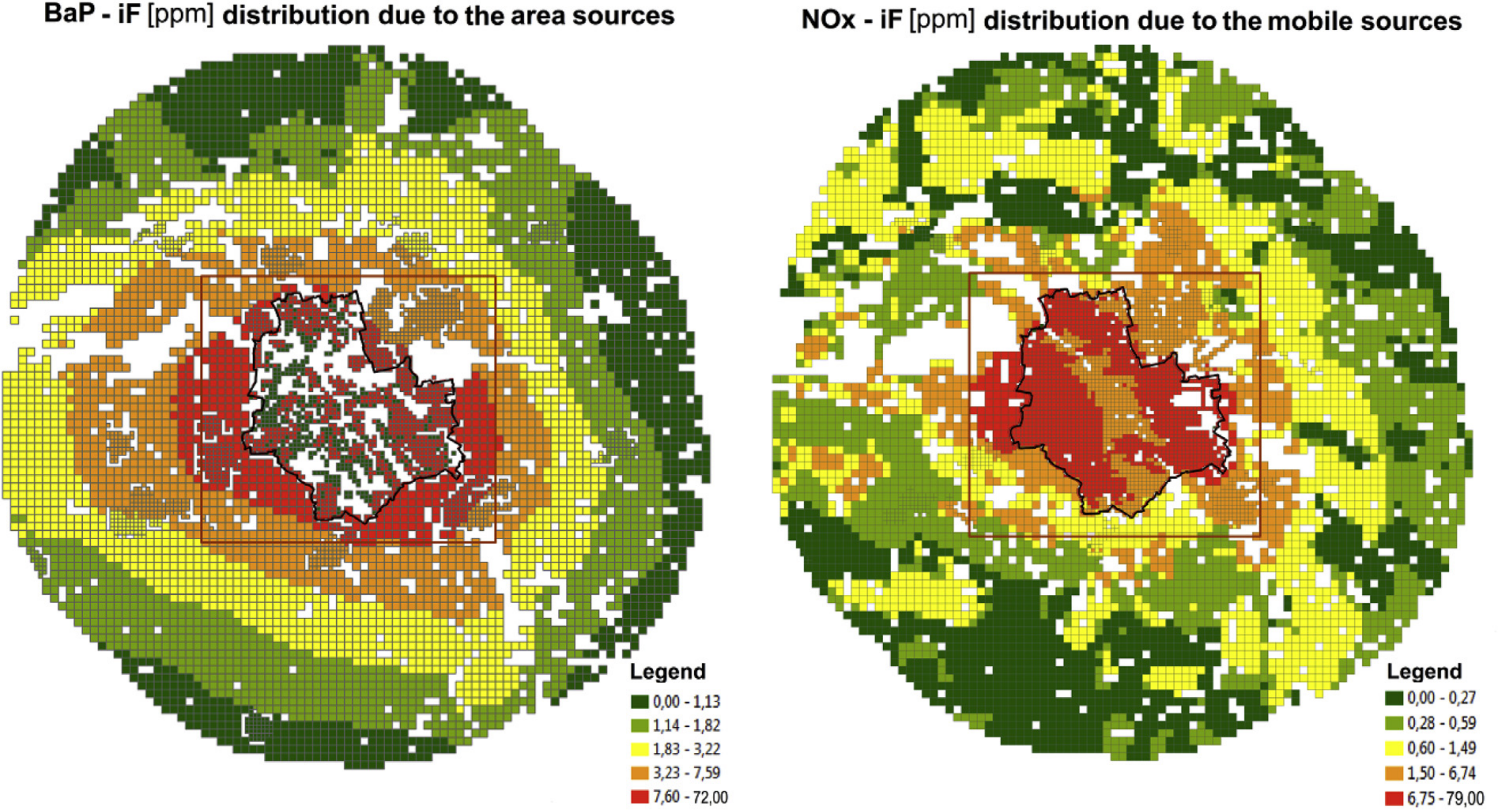
Spatial distributions of the *iF*s for the area sources (left) and the line sources (right).

Intra-urban variability of *iF*s attributed to single sources strongly depends on the emission domain that is taken into account. Fig. 3 (bottom) shows the *iF* distributions related to the area and the line sources for the total emission domain, while Fig. 4 shows the respective box plots for the Warsaw only domain. The highest *iF*s for the pollutants considered are the same, since they refer to the sources located inside Warsaw only (compare Fig. 5), but the IQRs are much wider, while the related medians and the upper whiskers raise significantly. The increase of IQRs due to change of the emission domain relates to all pollutants considered, as it is connected with abandoning of the more distant and mostly less impacting sources.

Fig. 5 presents maps of the spatial distribution of the *iF* estimates for the area (B(a)P) and the line (NO_X_) emission categories, respectively. For the area sources (Fig. 5, left panel), the maximum values occur inside the peripheral districts of Warsaw or in its close vicinity, where the location of the area sources (mainly residential combustion, with small scale heating installations) coincides with relatively high population density. The contribution of the area emission in the central part of the agglomeration is low (considerable part of the area with zero or negligible emission) due to the district heating in the main part of Warsaw.

**Fig. 6:**
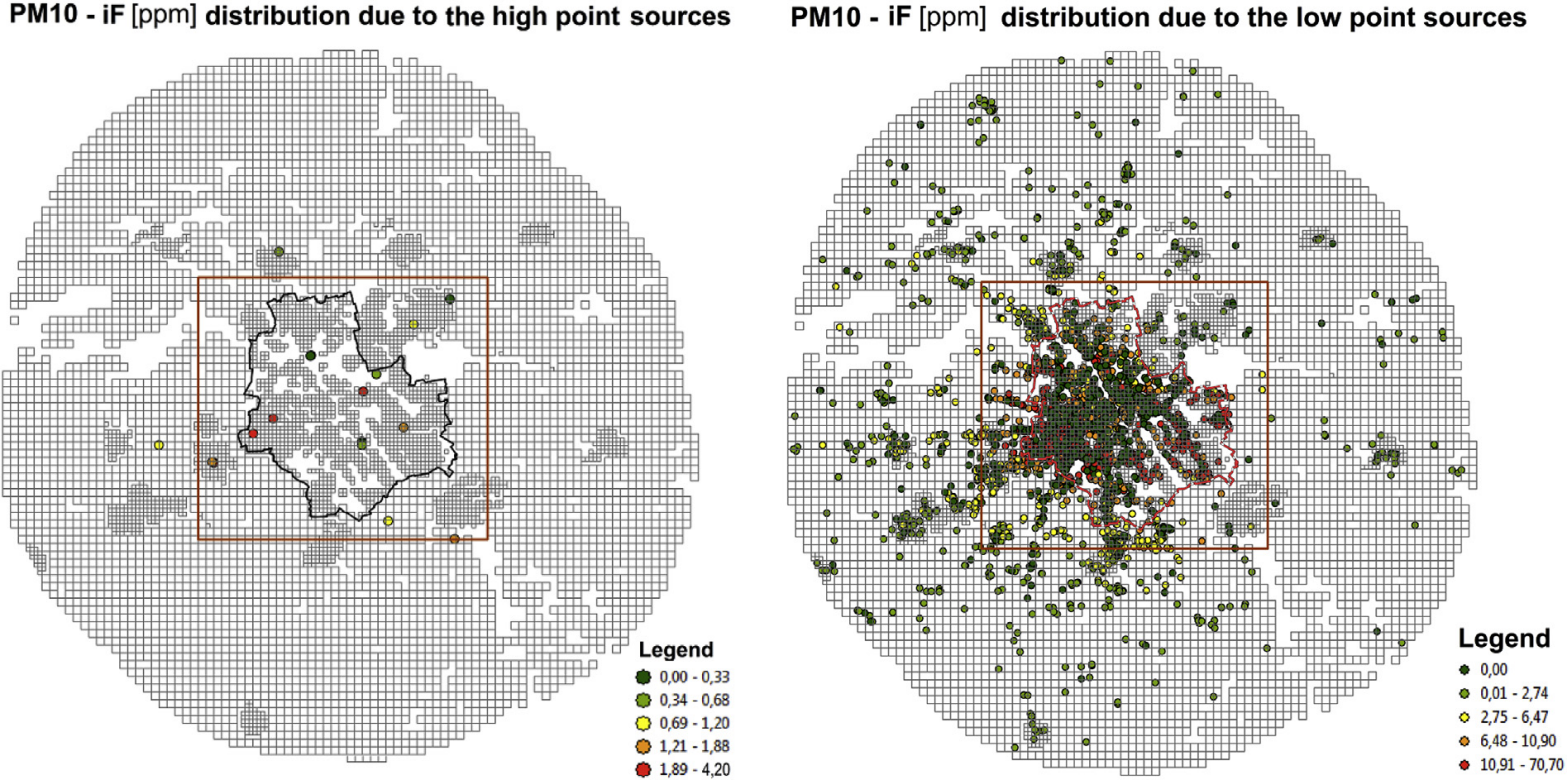
The *iF* estimates for the point sources: high (left) and low (right).

For the road transport sources, where *iF* distribution for NOx pollution is presented (Fig. 5, right panel), the highest *iF*s occur inside the Warsaw only domain, where the dense network of streets coincides with the high population density (with the distinct decrease in values along the channel of the Vistula river), cf. Fig. 1, Fig. 2. The area of the high *iF*s connected with the mobile sources is limited and much more compact compared to that for the area sources. Analogously, the spatial *iF* distribution maps for PM_10_ pollution in two categories of the point sources (the high point sources – left, and the low point sources – right) are presented in Fig. 6.

As stated above, *iF* is mainly related to such emitter's characteristics like its location, elevation of the emission point, population affected, meteorology etc., which depend neither on the pollutant nor emission volume. This fact is revealed by a very high correlation between *iF* values of different pollutants emitted by the same source. Since the atmospheric chemistry and removal of the secondary pollutants coming from the local sources have a minor impact, the *iF* value calculated for any selected pollutant characterizes in fact all the compounds emitted. Hence, each *iF* map presented in Fig. 5, Fig. 6 for a selected pollutant may be considered as representative for other polluting compounds in the same emission category.

### 3.3 Comparison of iFs and exposures

Due to the emission-normalized character of the *iF* metric, the impact of the emission rate on the resulting *iF* value is negligible, and no correlation between them is observed. On the other hand, the *iF* is very sensitive to the receptor area population density. This is illustrated by the above mentioned (Section 3.2), a specific low point source (represented by the maximum *iF*s for NOx and CO, seen in Fig. 3), where very low emission rate accompanied with high population density gives high value of *iF*.

**Fig. 7:**
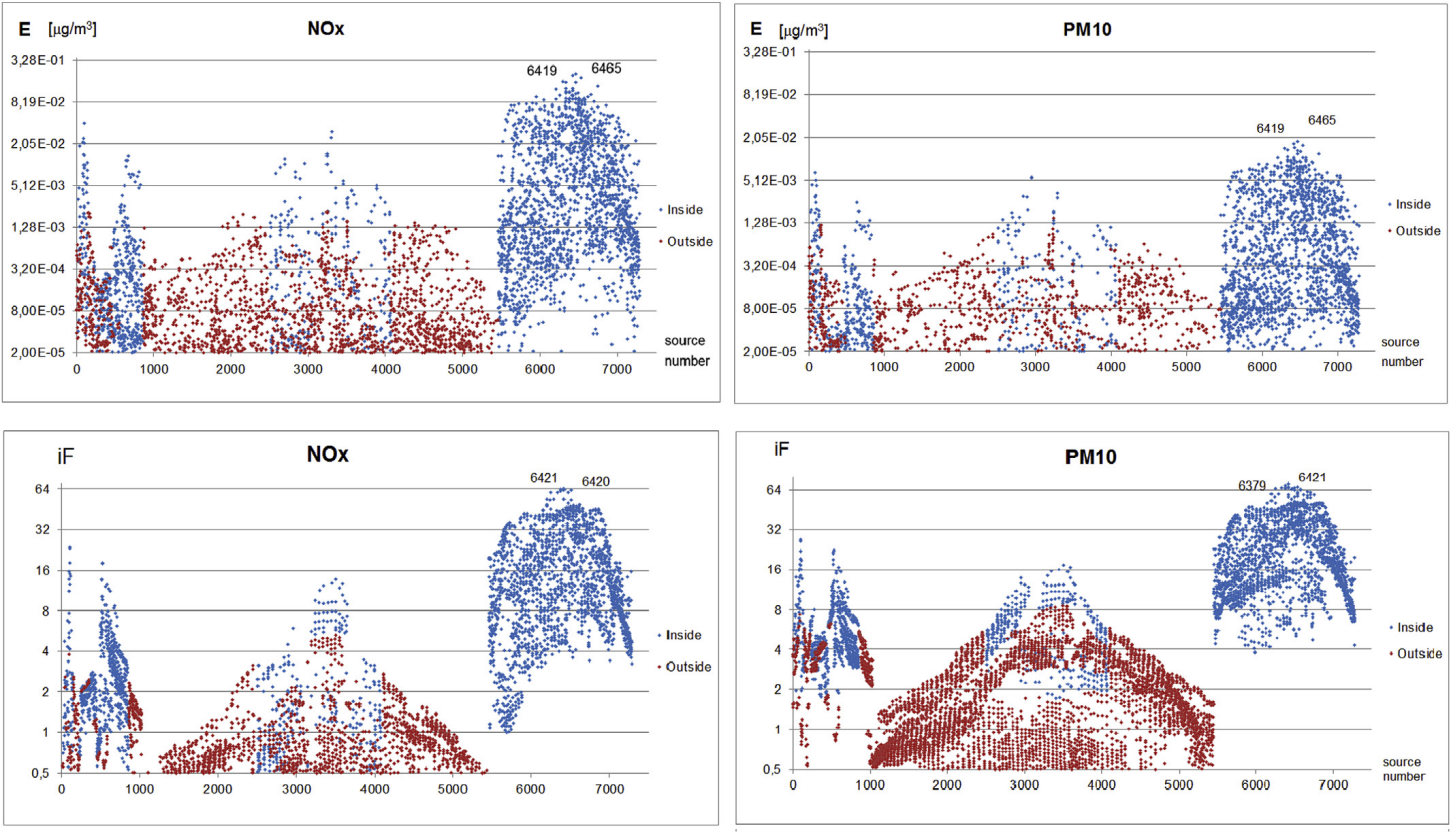
Spatial distributions of the exposure (*E*) and (*iF*) for NOx and PM_10_ for the line sources (logarithmic scale). Sources: #1-949 – cities outside Warsaw, 0.5 × 0.5 km resolution; #950-5499 – rural area outside Warsaw, 1 × 1 km resolution; #5500-7286 – inside Warsaw, 0.5 × 0.5 km resolution. Blue – sources located inside close vicinity of Warsaw (Fig. 1b), red – sources located outside this vicinity.

**Fig. 8:**
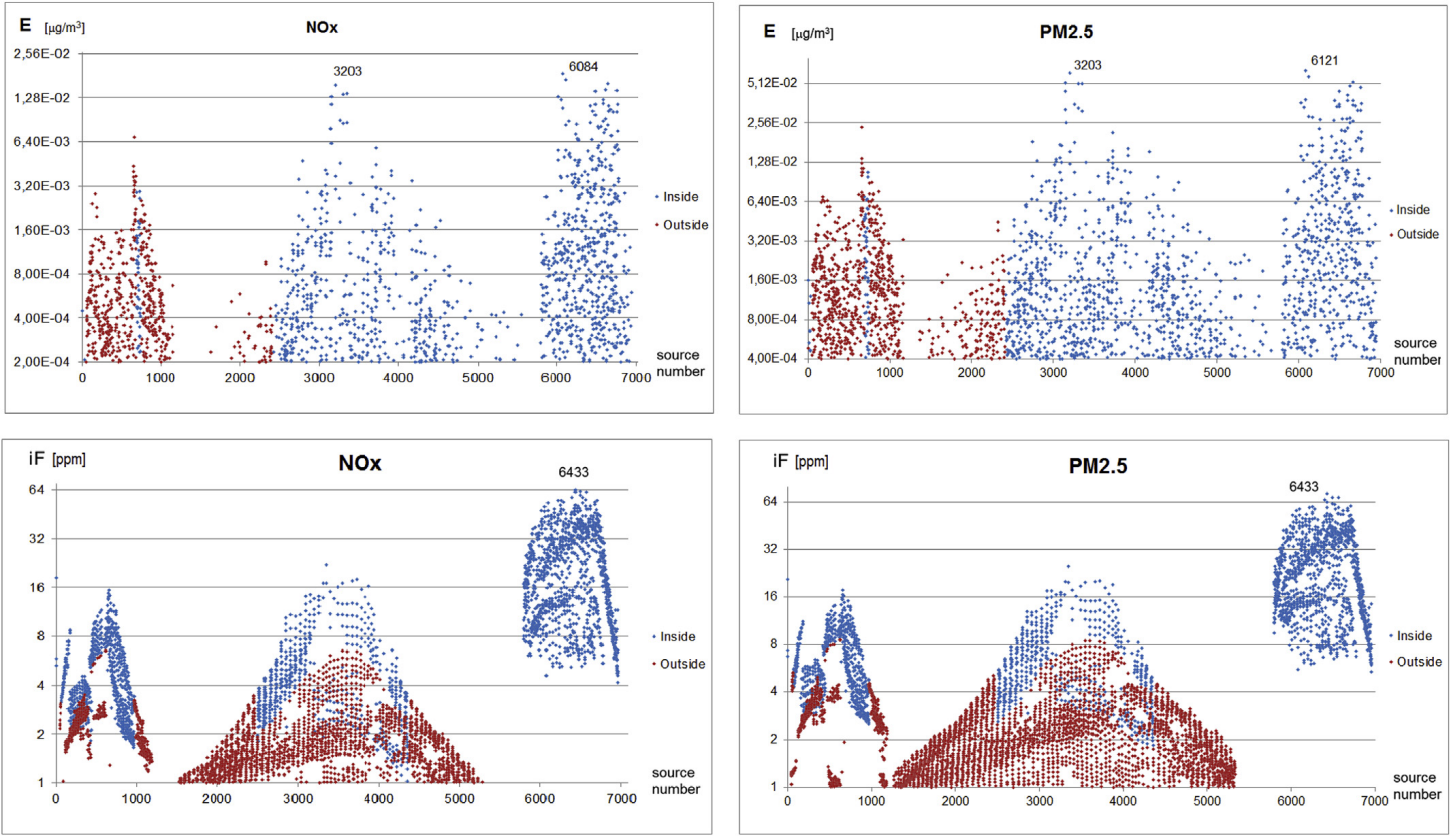
Spatial distributions of the exposure (*E*) and (*iF*) for NOx and PM_2.5_ for the area sources (logarithmic scale). Sources: #1-1100 – cities outside Warsaw, 0.5 × 0.5 km resolution; #1101-5800 – rural areas outside Warsaw, 1 × 1 km resolution; #5801-6963 – inside Warsaw, 0.5 × 0.5 km resolution. Blue – sources located inside close vicinity of Warsaw (Fig. 1b), red – sources located outside this vicinity.

Spatial distributions of *E* and *iF* for the mobile and the area sources are shown in Fig. 7, Fig. 8, respectively, where each dot represents a single emission source. For the line sources, #1–950 are the emitters in the cities surrounding Warsaw (fine grid), #950–5500 are the rural sources in the surroundings of Warsaw (coarse grid), and over #5500 are the sources located in Warsaw (fine grid). (Small differences in this subdivision of the area sources are explained in the caption of Fig. 8). Moreover, the distinction is marked between the sources located in Warsaw + close vicinity (blue color) and those outside it (red color).

Although both spatial distributions are similar, there are significant differences in the variability ranges of each of them, as shown in Fig. 7, Fig. 8, where the model scatter plots of both indexes are depicted for NO_X_ and PM_10_ emitted by the line, and NO_X_ and PM_2.5_ emitted by the area sources, respectively. About 2000 highest emission sources are considered in the plots. The logarithmic scale is used for Y-axis. The main difference is in the variability range of the *E* and *iF* plots. Values of *E* change in above three orders of magnitude, while those for *iF* only within the range 1–60 ppm. Flattening of the *iF* variability follows from its normalization by the emission rate, which increases *iF*s for relatively weak sources and reduces them for the strong ones. Hence, although the plot shapes are similar for *E* and *iF*, the highest value sources are different in each case. The sources with the highest exposures shown in Fig. 7, Fig. 8 (top), coincide with their maximal emissions. On the other hand, the highest *iF* values in Fig. 7, Fig. 8 (bottom) never coincide with the maximum emission sources. They rather reflect other factors mentioned above. This fact is also visible from the maximum value source numbers which are indicated in each plot for the indexes *E* and *iF*.

There are much more high *iF* mobile sources located in Warsaw only domain (Fig. 7), as most of the active area sources are located in the periphery of the city or in the city outskirt, due to the district heating system in the central part of Warsaw. On the other hand, Fig. S3 shows a significant contribution of the area sources from the Warsaw outskirt to *E* values, though it is partly caused by the 4 times increase of an emission cell area (1 km × 1 km).

### 3.4 Comparison to previous studies

The results of the present study are compared with several earlier *iF* estimates in Table 3. The earlier published results differ in the spatial scale of the region, method of analysis, basic emission category and pollutants analyzed. Thus, we mainly refer to the urban scale case studies and spatially distributed emission fields (mostly for the mobile sources). We use the aggregated *iF*s from the present study, estimated for the Warsaw only emission domain (Table 2).

**Table 3:** Comparison of the obtained intake fraction estimates (in ppm) with the earlier studies.

Reference	Region/pollution	Method	Reference *iF*			This study *iF*		
Tainio et al. (2014)	Warsaw urban area, domestic and traffic emissions (year 2005, resolution 1 × 1 km^2^)	CALPUFF model		a	t		a	t
			PM_10_	21	45	PM_10_	29	37
			PM_2.5_	20	44	PM_2.5_	28	39
			NOx	18	30	NOx	30	24
			SO_2_	18	32	SO_2_	30	26
Humbert et al. (2011)	Urban area – primary and secondary PM_2.5_ (ground-level emissions)	Expert group analysis		a/t			a	t
			PM_2.5_	44		PM_2.5_	28	39
			SO_4_	0.89		SO_4_	0.40	0.81
			NO_3_	0.18		NO_3_	0.13	0.60
Marshall et al. (2003)	Urban area (South Coast Air Basin, US) – traffic emissions of carbon monoxide and benzene	Monitoring data combined with time-activity patterns		t			a	t
			CO	32		CO	–	35
			C_6_H_6_	33		C_6_H_6_	–	35
Marshall et al. (2005)	Urban area – traffic emissions	One compartment method	21				a	t
						PM	–	39
						CO	–	36
						C_6_H_6_	–	35
Stevens et al. (2007)	Mexico City – ground level primary PM_2.5_	Five different methods compared	26–120			PM_2.5_ -- 39		
Loh et al. (2009)	Helsinki area - benzene from vehicles	EXPOLIS data	C_6_H_6_ 39			C_6_H_6_ 35		
Greco et al. (2007a).	Urban area (Boston) -- traffic, primary PM_2.5_	CAL3QHCR line source model	PM_2.5_ 8–50			PM_2.5_ 28		
Taimisto et al. (2011)	Country (Finland) -- traffic, primary PM_2.5_	Urban Dispersion Modeling system, FMI	PM_2.5_ 9.7			PM_2.5_ 39		
Greco et al. (2007b).	Across US. study – primary/secondary traffic PM_2.5_	Dispersion model -- the mobile emissions in urban areas		t			a	t
			PM_2.5_	9.8		PM_2.5_	–	39
			SO_4_	1.7		SO_4_	–	0.81
			NO_3_	0.23		NO_3_	–	0.60
Apte et al. (2012) Fantke et al. (2017)	Urban area - conserved pollutants from ground level emission	Global summary – one compartment method	am – 39 gm – 26				a	t
						PM	28	39
						CO	31	36
						C_6_H_6_	31	35

Abbreviations: a – area sources, t – traffic sources, am – arithmetic mean, gm – geometric mean.

In Tainio et al. (2014) study, the *iF* estimates for distributed and point sources in Warsaw are given, using the CALPUFF model with the spatial resolution 1 × 1 km and the input data for the year 2005. An increase of *iF* estimates for the area sources that is observed in our results is due to new residential investments in the recent years, mainly in the peripheral districts. Despite different spatial resolutions applied in both studies, and much higher number of emission sources considered now, the *iF* estimates for the line sources are quite close each other (20 ppm and 28 ppm for PM2.5, in Tainio et al. and this study, respectively).

Humbert et al. (2011) summarize the work of an international expert group to analyze the primary and secondary ground-level emissions of PM_2.5_ in an urban area. Results are comparable with the traffic related *iF*s in our study. Estimates obtained by Marshall et al. (2003) for CO and C_6_H_6_ from traffic emissions in South Coast Air Basin in US (calculated for the monitoring data combined with time-activity patterns) are also close to the respective *iF*s in our study. In (Marshall et al., 2005) the mean *iF* for urban traffic emissions (21 ppm) obtained by one-compartment modeling is lower than our estimates, but they apply lower breathing rate (12.2 m^3^d^-1^). When updating their *iF* value with our breathing rate of 20 m^3^d^-1^, very close value of 34.4 ppm is obtained. Stevens et al. (2007) compare five methods to estimate *iF* for PM_2.5_ in Mexico City. The resulting values range from 26 ppm (regression) up to 120 ppm (steady state box model) versus our 39 ppm. However, it must be noted that Mexico City is classified as a megacity with several million inhabitants and high population density (Apte et al., 2012), much different from the Warsaw conditions. Intake fraction estimate by Loh et al. (2009) for benzene emitted by vehicles in the Helsinki urban area (39 ppm) is very close to our result for the line sources (35 ppm). Greco et al. (2007a) apply an urban scale, line source model to analyze the primary PM_2.5_ emissions in the Boston Urban Area.

On the other hand, an aggregated results for PM_2.5_ emissions in Finland (Taimisto et al., 2011) are lower than the corresponding estimates in this study, which is not surprised considering population density differences between Finland and Warsaw. Greco et al. (2007b) apply a dispersion model to analyze the primary and secondary PM_2.5_ emissions across the United States. The resulting *iF*s in their study are the averages from 19 agglomerations, and the estimate for the primary PM_2.5_ is similar to that from Taimisto et al. (2011). A different modeling approach is applied by Apte et al. (2012) and Fantke et al. (2017), where they apply one-compartment method for global analysis of ground-level emissions in 3646 cities with the total population of 2 billion. Their mean iF values attributed to the conserved primary pollutants, for the cities of the Warsaw's scale, are comparable with those of the present study.

## 4 Concluding remarks

In general, the most important factors influencing the intake fraction are, as stated above, source location (distance from the receptor domain in our case), urban morphology, density and size of the population exposed, meteorological conditions, pollutant persistence and transformations. The elevation of the effective emission point is another important factor (Fig. 3, Table 2). As a rule, *iF* values (mean, maximum, IQR) rise from high-, to low-point, area, and line sources, respectively. The *iF* estimates are defined for the unit emission rate, hence they do not depend on the emission strength. Small emitters, like the specific source mentioned above (Section 3.2), when emitting at a low height and located in a densely populated area, may have a high *iF* value. Those with high stacks have small *iF* values, even if their emission is high. Strong correlations are observed between *iFs* of the primary pollutants emitted by the same source, as mentioned above in Section 3.2. On the other hand, similar or identical sources with different locations may substantially differ in *iF* values, mainly due to a strong impact of the spatially variable population density.

Urban morphology may impact the actual values of intake fractions. For the cities with compact building developments, street canyons influence quite considerably distribution of the population exposures to air pollutants, particularly the traffic-related ones (Zhou and Levy, 2008; Hang et al., 2017). However, Warsaw has rather a dispersed building developments with wide main streets and many open areas. Hence, any more significant corrections of the estimated intake fractions may apply only to limited areas of the city.

Another factor that can influence the results is variability of the breathing rate among groups of inhabitants, like cyclists versus pedestrians or children versus adults. Corrections due to this factor would necessitate very detailed analysis of e.g. bicycle traffic, localization of kindergartens and schools, etc. Average breathing rate seems to be a good approximation in this case.

Spatial distributions of *iFs* for individual sources in four emission categories, illustrated on maps in Fig. 5, Fig. 6, are quite characteristic for the area and the line emitters. The highest *iFs* of the area category are connected with the sources located in the peripheral districts of Warsaw or in the close neighborhood (domestic heating). Very low (or zero) values in the central part of the city are the consequence of the district heating there. On the other hand, the domain of the highest *iFs* for the line sources is compact and limited, and covers the central districts, where high population density coincides with high traffic intensity. Although the maps in Fig. 5 refer to BaP (area sources) and NOx (line sources), respectively, the maps are representative for any primary pollutant within the same emission category, and the corresponding maps for other pollutants in the same emission category are almost identical. This is an effect of high correlation between the pollutants emitted by the same source. Hence, a representative *iF* estimate for one pollutant can be sufficient for quick inspection of the source importance.

The urban population health risk depends highly on pollutant types. Final decision concerning emission policy is however constrained by economic factors, as usually the funds of the abatement projects are limited. Some results of this study suggest that reduction of emissions from suitably chosen small sources with high exposure risk may give even better effects than reduction of emissions from big sources, if economically sound. So, in final abatement planning, projects connected with reduction of small sources emissions should not be a priori ignored in optimization.

## Conflict of interest statement

The authors certify that they have no conflict of interest concerning the subject matter or materials discussed in the manuscript.
